# Expansion of Coccidioidomycosis Endemic Regions in the United States in Response to Climate Change

**DOI:** 10.1029/2019GH000209

**Published:** 2019-10-10

**Authors:** Morgan E. Gorris, Kathleen K. Treseder, Charles S. Zender, James T. Randerson

**Affiliations:** ^1^ Department of Earth System Science University of California Irvine CA USA; ^2^ Department of Ecology and Evolutionary Biology University of California Irvine CA USA

**Keywords:** Coccidioidomycosis, Valley fever, mycoses, infectious diseases, human health, niche model

## Abstract

Coccidioidomycosis (Valley fever) is a fungal disease endemic to the southwestern United States. Across this region, temperature and precipitation influence the extent of the endemic region and number of Valley fever cases. Climate projections for the western United States indicate that temperatures will increase and precipitation patterns will shift, which may alter disease dynamics. We estimated the area potentially endemic to Valley fever using a climate niche model derived from contemporary climate and disease incidence data. We then used our model with projections of climate from Earth system models to assess how endemic areas will change during the 21st century. By 2100 in a high warming scenario, our model predicts that the area of climate‐limited endemicity will more than double, the number of affected states will increase from 12 to 17, and the number of Valley fever cases will increase by 50%. The Valley fever endemic region will expand north into dry western states, including Idaho, Wyoming, Montana, Nebraska, South Dakota, and North Dakota. Precipitation will limit the disease from spreading into states farther east and along the central and northern Pacific coast. This is the first quantitative estimate of how climate change may influence Valley fever in the United States. Our predictive model of Valley fever endemicity may provide guidance to public health officials to establish disease surveillance programs and design mitigation efforts to limit the impacts of this disease.

## Key Points

- We created a niche model to estimate climate limits on the spatial extent of Valley fever endemicity in the United States
- For a high warming scenario, the area of climate‐limited endemicity will more than double by 2100, expanding northward into dry western states
- Our predictive model of Valley fever endemic regions may help mitigate disease impacts as the disease spreads into new regions

## 1 Introduction

Coccidioidomycosis, commonly known as Valley fever, is an infectious fungal disease that has gained attention in the United States due to a recent increase in cases (Centers for Disease Control and Prevention (CDC), 2018a). Humans contract Valley fever when they inhale *Coccidioides* spp. fungal spores. At onset, symptoms of Valley fever closely resemble the flu, which may delay diagnosis (CDC, 2018b). If left untreated, debilitating symptoms may occur, and on rare occasion may cause death. Valley fever is not a communicable disease, so cases are a result of human exposure to *Coccidioides* spp. in the environment.

*Coccidioides* spp., and therefore Valley fever, is endemic to the southwestern United States and parts of Central and South America (CDC, 2017). Currently, there are two known species of *Coccidioides*, both of which cause Valley fever: *C. immitis* and *C. posadasii* (Lauer, 2017). *C. immitis* is thought to be the primary species present in California, while *C. posadasii* has a broader geographic distribution and is more commonly found in the highly endemic areas of Arizona (Barker et al., 2019; Lauer, 2017). The fungi grow as hyphae within desert soils (Stewart & Meyer, 1932). As such, *Coccidioides* spp. growth and abundance are influenced by environmental conditions (Maddy, 1957). The fungi proliferate during wet periods. When water becomes limiting, *Coccidioides* spp. hyphae then break apart into spore‐containing fragments, small enough for humans to inhale (Maddy, 1957). Any type of soil disturbance, like high winds or digging in dry soils, can cause *Coccidioides* spp. spores to become airborne and potentially inhaled by humans. Many details about the *Coccidioides* spp. life cycle and the microecosystem characteristics that structure its presence in soils are unknown. As a consequence, environmental surveillance for the fungi has yielded relatively few soil samples that have tested positive for *Coccidioides* spp.

Because the fungi have not been systematically mapped across the hypothesized endemic region, much of our understanding of the relationships between environmental factors and *Coccidioides* spp. comes from studying epidemiological data. On a regional scale, weather and climate are known to influence the seasonal and interannual variability of disease incidence. Previous studies support a pattern of wet, then dry conditions preceding increased Valley fever incidence across the southwestern United States (Comrie, 2005; Coopersmith et al., 2017; Gorris et al., 2018; Kolivras & Comrie, 2003; Komatsu et al., 2003; Park et al., 2005; Talamantes et al., 2007; Tamerius & Comrie, 2011; Zender & Talamantes, 2006). These dual controls first increase fungal growth during periods of higher than normal moisture. Then, they increase spore production and effective dispersal when hot temperatures and low rainfall desiccate soils and enhance the production of dust. Time delays between drying and elevated levels of incidence are observed in the two highly endemic regions, the San Joaquin Valley of California and south‐central Arizona, despite regional differences in the timing of precipitation (Gorris et al., 2018). On finer temporal and spatial scales, processes such as soil disturbance, dust storms, and agricultural activity can also influence Valley fever incidence (Tong et al., 2017; Wilken et al., 2015; Williams et al., 1979).

These connections between climatic conditions and disease dynamics suggest that on regional scales, climate may also structure the environmental range of the fungi, and therefore, the spatial extent of Valley fever endemicity (Baptista‐Rosas et al., 2007; Fisher et al., 2007). Two main climate conditions that regulate the occurrence of *Coccidioides* spp. in the environment are temperature and precipitation (Baptista‐Rosas et al., 2007; Fisher et al., 2007; Gorris et al., 2018). County‐level Valley fever case reports from 2000 to 2015 across five states in the southwestern United States revealed the spatial pattern of incidence has a nonlinear positive relationship with mean annual temperature and nonlinear inverse relationship with mean annual precipitation (Gorris et al., 2018). Ultimately, these two climate conditions structure the presence of deserts: the biome in which *Coccidioides* spp. thrives (Fisher et al., 2007; Maddy, 1957). High temperatures may limit the growth of many microbial competitors, allowing *Coccidioides* spp. to more effectively compete for soil resources (Barker et al., 2012; Greene et al., 2000). Low levels of precipitation in deserts may also limit microbial competitors; however, occasional periods of high moisture availability are important for *Coccidioides* spp. fungal growth and reproduction (Fisher et al., 2007; Maddy, 1957). In contrast, wet soils in regions with high mean annual precipitation may limit dust production, spore dispersal, and thus human exposure to *Coccidioides* spp. (Gorris et al., 2018).

There is also preliminary evidence from the few soil samples positive for *Coccidioides* ssp. that temperature and precipitation may be important for structuring the spatial pattern of Valley fever endemicity. Most soil samples positive for *Coccidioides* ssp. were collected from the Central Valley of California (Colson et al., 2017; Lauer et al., 2012, 2014), south‐central Arizona (Barker et al., 2012), and Baja Mexico (Baptista‐Rosas et al., 2012; Catalán‐Dibene et al., 2014; Vargas‐Gastélum et al., 2015)—all areas that are hot and dry. Nineteen soil samples positive for *Coccidioides* ssp. and measures of both temperature and precipitation along with a large suite of other bioclimatic variables were used in the first known statistical environmental niche model of *Coccidioides* ssp. in northwestern Mexico and parts of the southwestern United States (Baptista‐Rosas et al., 2007). This model identified the most likely habitat for *Coccidioides* spp. as the Lower Sonoran Desert and successfully highlighted epidemiological hotspots of Valley fever in the Central Valley of California and south‐central Arizona. However, the niche model derived from this set of observations cannot fully explain the current spatial pattern of Valley fever cases (CDC, 2018b). This may be a consequence of the relatively small number of soil samples used to initialize the model. Until soils are systematically sampled across the western United States, epidemiological data may provide a more robust way of delineating the effects of temperature and precipitation on the regions endemic for Valley fever.

Climate change is increasing temperatures and shifting precipitation patterns throughout the United States. These changes could alter the regions endemic to Valley fever, as well as the number of Valley fever cases. Temperatures in the contiguous United States are expected to increase by 1.6–6.6°C by 2100 (relative to 1986–2015) under a high greenhouse gas emissions scenario, representative concentration pathway 8.5 (RCP8.5; Hayhoe et al., 2018). This warming may allow *Coccidioides* spp. to expand its geographical range farther north, in areas previously unsuitable for the species to survive. Precipitation projections are more uncertain for the western United States, and changes will likely vary by region and season (Easterling et al., 2017; Hayhoe et al., 2018; Swain et al., 2018). Along the Pacific coast, especially in the Pacific Northwest, mean annual precipitation is projected to increase (Easterling et al., 2017; Hayhoe et al., 2018). In contrast, the southwestern United States will likely experience little to no change in precipitation, while the southern Great Plains may become drier. In dry areas, increasing temperatures will likely increase evaporative demand, which may contribute to desertification. The expansion of dryland ecosystems may increase the area suitable for the occurrence of *Coccidioides* spp., along with the production of dust and fungal spores.

To predict how climate change may influence the spatial pattern of Valley fever in the future, it is important to have an accurate map of the current endemic area. The basis of the U.S. Centers for Disease Control and Prevention (CDC) estimate of endemic areas is a historical skin test study of approximately 88,000 young men at a Naval Training Center in San Diego, CA, from 1948 to 1950 that detected exposure to *Coccidioides* spp. (CDC, 2018b; Edwards & Palmer, 1957). Since the original study, the endemicity map has been modified to account for localized outbreaks of Valley fever (Marsden‐Haug et al., 2012; Petersen et al., 2004; Werner et al., 1972; Werner & Pappagianis, 1973). One outbreak caused by *C. immitis* occurred in Washington State, well outside its normal geographical range in the Central Valley of California and outside the hypothesized endemic region of Valley fever throughout the southwestern United States (Litvintseva et al., 2014; Marsden‐Haug et al., 2012).

More recently, a county‐level map of mean annual Valley fever incidence derived from 16 years of epidemiological data collected from state health agencies provided an independent way to estimate endemic areas (Gorris et al., 2018). This incidence database has not been used with niche modeling to explore the spatial pattern of disease. Valley fever case reports alone may be an underestimate of the actual burden of disease due to misdiagnosis, underreporting, or other host factors (Ampel, 2010; Chang et al., 2008; Jones et al., 2017). Despite this limitation, further analysis of epidemiological data may provide a means to better estimate where Valley fever is currently endemic. This could allow public health agencies to improve surveillance programs and help decrease the time to patient diagnosis. The incidence database also provides a starting point for predicting how climate change will modify the location and extent of endemic areas.

The goal of our work was to create a model that describes the area in the United States currently endemic to Valley fever and then to use this model to predict how the endemic area may shift in response to climate change. First, we used established relationships between climate and the spatial distribution of Valley fever incidence to create a climate‐constrained niche model that predicts the contemporary pattern of Valley fever endemicity. Then, we used our niche model with climate projections from Earth system models to analyze where the climate limits are lifted in the future, potentially allowing the area to become endemic to Valley fever. A secondary goal of our work is to estimate future Valley fever incidence and the number of people who may contract this disease. We report future estimates of the endemic area and potential changes in incidence for the years 2035, 2065, and 2095 under both moderate and high climate warming scenarios. We also examine the compounding effect of climate change and increases in human population on the number of people living in the endemic region and number of potential Valley fever cases. This is the first quantitative projection for the United States of how climate change may affect Valley fever. Our predictive model of the endemic area to Valley fever and estimate of future disease burden may provide guidance to public health officials as to where increased Valley fever surveillance and education may improve health outcomes.

## 2 Methods

### 2.1 Valley Fever Incidence Data

To create our models of endemic area and incidence, we used a previously compiled data set of Valley fever cases for the southwestern United States (Gorris et al., 2018). This data set included monthly, county‐level Valley fever cases from 2000 to 2015 from Arizona, California, Nevada, New Mexico, and Utah. We calculated Valley fever incidence for each county using 2000–2015 intercensal population estimates from the U.S. Census Bureau (U.S. Census Bureau, 2011, 2016). We performed our analysis at the county‐level, which was the highest resolution available from the state health agencies for the deidentified, aggregated case data.

### 2.2 Current and Future Projections of Climate

We focused our analyses on two main climate drivers that influence the presence of *Coccidioides* ssp. in the environment: temperature and precipitation. We gathered surface air temperature and surface precipitation data for years 2000–2015 to compare directly with Valley fever incidence. For both climate variables, we used 4‐km gridded products from the Precipitation‐elevation Regressions on Independent Slopes Model (PRISM; Daly et al., 2008). To compare county‐level Valley fever incidence data with climate data, we calculated county‐level climate averages by spatially averaging the gridded PRISM climate data within each county using QGIS (https://www.qgis.org). We obtained county shapefiles from the U.S. Census Bureau (https://www.census.gov/geo/maps‐data/data/tiger‐line.html).

**Figure 1:**
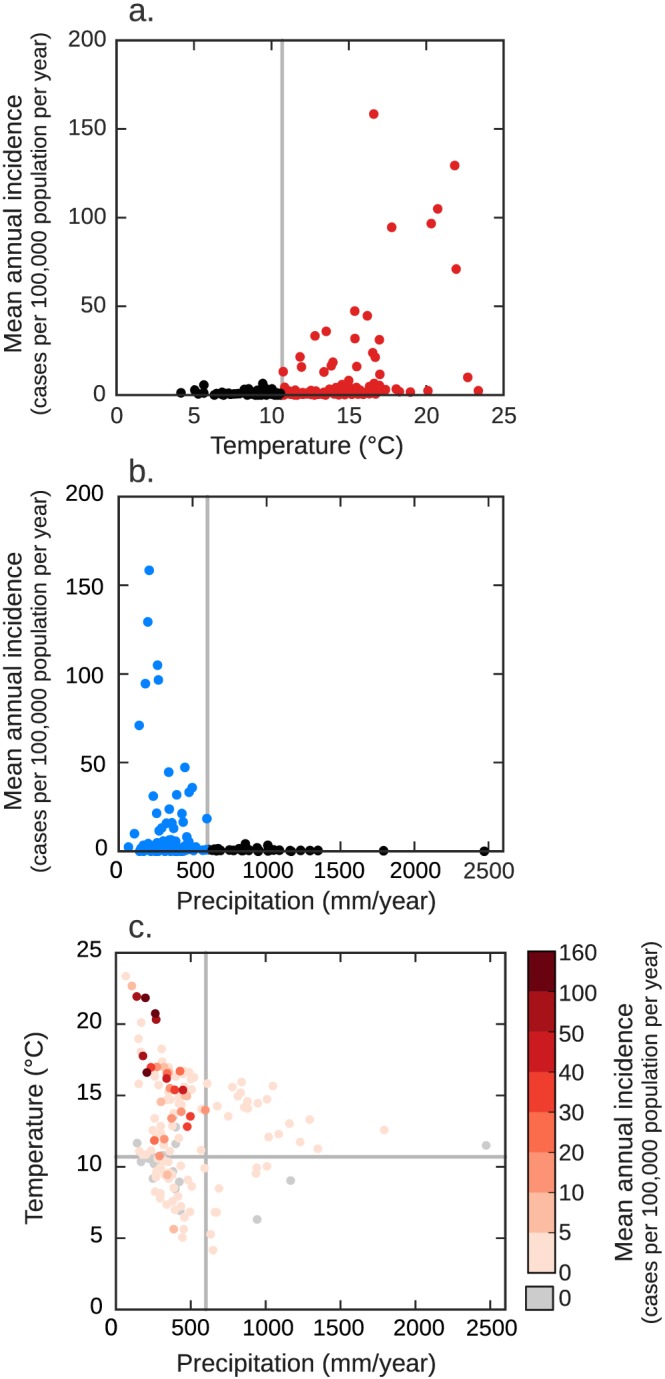
Valley fever incidence for counties in the southwestern United States (*n* = 152) as a function of (a) mean annual temperature and (b) mean annual precipitation. All counties that have endemic levels of Valley fever incidence (defined as meeting or exceeding 10 or more cases per 100,000 population per year during 2000–2015; *n* = 23) have a mean annual temperature greater than or equal to 10.7°C and a mean annual precipitation level less than or equal to 600 mm/year. (c) Counties with higher levels of mean annual Valley fever incidence are concurrently hotter and drier. We adapted panels a and b of this figure from Gorris et al. (2018) and added the gray lines to indicate the position of the climate thresholds we used to build our climate‐constrained niche model.

In previous work, we found a significant, positive nonlinear relationship between county‐level mean annual temperature and Valley fever incidence, and a significant, nonlinear inverse relationship between mean annual precipitation and incidence throughout the southwestern United States (Figures 1a and 1b; Gorris et al., 2018). We previously analyzed a suite of climate variables and found that mean annual temperature, precipitation, soil moisture, surface dust concentration, and county cropland area had significant relationships with the spatial distribution of Valley fever incidence in the southwestern United States (Gorris et al., 2018). We chose to use precipitation here instead of soil moisture for mapping the spatial extent of Valley fever because of large model‐to‐model differences in the representation of the processes regulating soil moisture content in the Coupled Model Intercomparison Project Phase 5 (CMIP5) models. We did not include dust or cropland area because of the ineffectiveness of these variables in constraining Valley fever endemic areas at the continental scale of the contiguous United States (data not shown). Analysis of these data shows that counties with higher levels of mean annual Valley fever incidence have a hot and dry contemporary climate (Figure 1c).

For future climate projections, we used output of monthly surface air temperature (variable “tas”) and surface precipitation (variable “pr”) from 30 Earth system models from the Bias‐Corrected Spatially Downscaled CMIP5 Climate Projections archive (Table S1 in the supporting information; available at https://gdo‐dcp.ucllnl.org/downscaled_cmip_projections/; Maurer et al., 2007; Reclamation, 2013). The CMIP5 model simulations were used extensively in the Intergovernmental Panel on Climate Change 5th Assessment Report (Stocker et al., 2013; Taylor et al., 2012). We analyzed data for RCP4.5, a moderate fossil fuel emissions and warming scenario in which emissions peak in the mid‐21st century and decrease thereafter, and RCP8.5, a high fossil fuel emissions and warming scenario in which emissions increase continuously through the 21st century (Moss et al., 2010). We calculated a mean annual temperature for our analyses by averaging the raw, gridded monthly temperatures, and we calculated a mean annual precipitation by taking the sum of monthly precipitation for each year, separately for each of the 30 models.

To estimate future climate, we combined information from the CMIP5 simulations with contemporary climate observations from PRISM. We first selected a baseline period of 2007 (averaging across the years 2000–2015) to match the period of available Valley fever case data. We averaged the raw, gridded CMIP5 output to calculate future mean annual temperature and precipitation for 2035 (the average of years 2030–2040), 2065 (the average of years 2060–2070), and 2095 (the average of years 2090–2100). We used 11‐year averages to reduce (but not eliminate) the uncertainty associated with low‐frequency internal variability that can make it difficult to detect or quantify trends from anthropogenic forcing (Deser et al., 2012). Next, we spatially averaged these climate projections to the county‐level. We then calculated climate anomalies as the difference between each of these future time periods and our baseline period for each county, separately for each CMIP5 model. For mean annual temperature, we calculated the absolute difference between our baseline and each future time period. For mean annual precipitation, we calculated the percent change between the baseline and each future time period. We created climate projections by adding the CMIP5 climate anomalies to our 2007 baseline PRISM data. We averaged the climate anomalies from the 30 CMIP5 simulations to create a multimodel mean climate projection that we added to our 2007 baseline PRISM data; we used this multimodel mean to create our main projections of Valley fever endemicity.

To provide an estimate of the uncertainty in our multimodel mean, we calculated the standard deviation across the 30 CMIP5 simulations for each Valley fever statistic. As another measure of climate projection uncertainty, we report individual model projections of the number of counties endemic to Valley fever in 2095 for both RCP4.5 and RCP8.5 climate scenarios in Table S1. We further used the individual CMIP5 simulations to quantify the level of agreement among models that each county will become endemic to Valley fever for both the RCP4.5 and RCP8.5 scenarios and report this uncertainty metric as a map.

**Figure 2:**
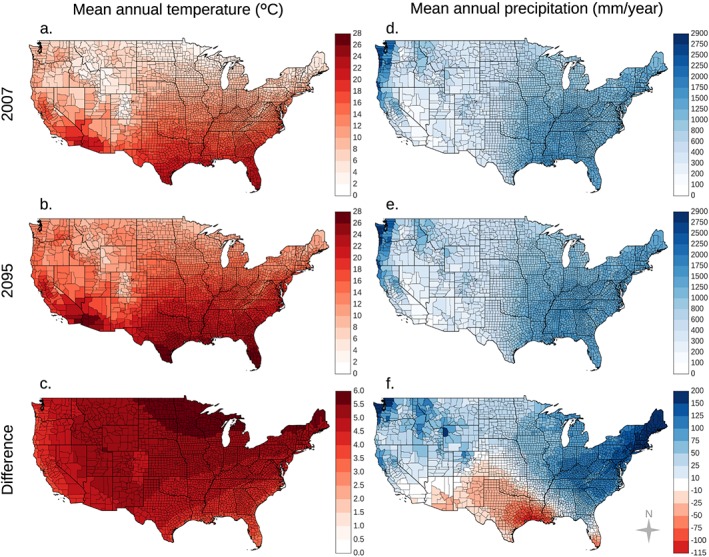
Representative concentration pathway (RCP) 8.5 climate projections indicate warming throughout the contiguous United States with the highest levels occurring in northern states (a–c). Changes in precipitation will vary by region (d–f). RCP8.5 projections indicate drying in the southwestern United States and south‐central Great Plains and wetting across the Pacific Northwest and eastern United States. The difference panels (c and f) are the difference between the 2095 and 2007 maps for each climate variable.

Our climate projections show the highest warming in the north‐central contiguous United States and relatively high levels of warming throughout the northern United States and Rocky Mountains (Figures 2a–2c and S1 in the supporting information). Mean annual temperatures are predicted to increase for the RCP8.5 climate scenario by 3.1 to 6.0°C by the end of the 21st century. Mean annual precipitation is predicted to increase across the Pacific Northwest and in the eastern United States but decrease in the south‐central and southwestern United States (Figures 2d–2f and S2). Both the increase in temperature and changes in precipitation are larger for the RCP8.5 climate scenario than for the RCP4.5 climate scenario (Figures S1 and S2).

### 2.3 Climate Niche Modeling of Current and Future Valley Fever Endemic Regions

We used the observed relationships between mean annual Valley fever incidence and both mean annual temperature and precipitation to map regions endemic to Valley fever. First, we selected a minimum level of mean annual Valley fever incidence (averaged from 2000 to 2015) to designate that a county was endemic by comparing our Valley fever incidence data against the CDC endemicity map. We found that there were large variations in the mean annual incidence between the three CDC definitions of endemicity: counties considered “highly” endemic by the CDC had mean annual Valley fever incidence between 21.3 and 158.4 cases per 100,000 population per year. Counties considered “established” endemic had between 0.7 and 94.5 cases per 100,000 population per year. Counties considered “suspected” endemic had between 0.0 and 31.8 cases per 100,000 population per year.

Taking these large variations into account, we selected a conservative level of mean annual incidence to define a county as endemic, which we defined as meeting or exceeding 10 cases per 100,000 population per year. This definition included all the counties the CDC defined as “highly” endemic (5/5), over half the counties the CDC described as “established” endemic (16/28), and one county the CDC described as “suspected” endemic (1/44; San Luis Obispo, CA; mean annual incidence of 31.8 cases per 100,000 population per year).

Then, we examined the mean annual temperature and precipitation for the counties we defined as endemic. For temperature, all of the counties we defined as endemic have a mean annual temperature above 10.7°C (Figure 1a). For precipitation, all of the counties we defined as endemic have mean annual precipitation less than 600 mm/year (Figure 1b). We used these two thresholds together to create a climate‐constrained niche model, which describe the climate necessary for Valley fever endemicity. Our niche model identifies a county as endemic if that county has both a mean annual temperature greater than or equal to 10.7°C and mean annual precipitation less than or equal to 600 mm/year (Figure 1c).

We applied our climate‐constrained niche model to the entire United States to estimate the areas which may currently be endemic to Valley fever. Then, we used our climate projections for both the RCP4.5 and RCP8.5 scenarios as input to estimate the areas that may become endemic to Valley fever in years 2035, 2065, and 2095.

We attempted different optimizations of our incidence and climate thresholds to improve the accuracy of our map in comparison to the CDC map. When we apply our climate‐constrained niche model to the United States, we acknowledge that there may be differences between the area we defined as endemic and the area the CDC defines as endemic since the basis for the CDC map is over 65 years old (Edwards & Palmer, 1957). Moreover, Valley fever incidence varies widely for counties within each of the three classes of endemicity defined by the CDC.

As a sensitivity analysis to complement our climate‐constrained niche model, we ran the ecological niche model Maxent (R package “dismo” version 1.1‐4 and Maxent version 3.3.3k; Phillips et al., 2006). We trained our model by defining occurrence points as the counties that met our definition of endemicity (10 cases per 100,000 population per year; *n* = 23). All other counties were considered background points (*n* = 3085). We ran our models with default configurations, so all feature types were possible. We ran two scenarios with Maxent: one with the PRISM baseline mean annual temperature and mean annual precipitation as explanatory variables and a second with the PRISM mean January temperature, mean July temperature, and mean annual precipitation as explanatory variables. The output of Maxent is a relative environmental suitability measure, ranging from 0 to 1, where 1 describes an environment most similar to the training data set. To identify a county‐level endemicity threshold, we optimized the environmental suitability variable to attain the highest accuracy when compared to the CDC endemicity map (comprised of all three CDC endemicity classes; Table S2). Counties at or above this suitability threshold were considered endemic. After this optimization, the two‐variable Maxent model has an accuracy of 96.3% and the three‐variable Maxent model has an accuracy of 96.8%. As described below, we compared the areas identified as endemic to Valley fever by our climate‐constrained niche model to the more conservative predictions from the Maxent models as a sensitivity analysis and report the results in Table S2.

### 2.4 Modeling of Current and Future Mean Annual Valley Fever Incidence

We estimated an upper bound of current and future for counties our climate‐constrained niche model defined as endemic. To do so, we applied a multiple linear quantile regression using iterative reweighted least squares for the 90th percentile using the observed relationships between mean annual Valley fever incidence (*VFI)* and mean annual temperature (*T*) and precipitation (*P*) for the endemic counties (red and blue colored counties in Figure 1, *n* = 78).

$$\begin{array}{l} \mathrm{VFI}=\beta_{1}T+\beta_{2}P\\ \mathrm{VFI}=\left(6.57\right)T+\left(-0.12\right)P \end{array}$$

Our model had a pseudo *r*‐square (not analogous to ordinary least squares *r*‐square) value of 0.29 describing the local fit for our baseline period. We chose to report the 90th percentile estimate as an indicator of potential Valley fever incidence, recognizing that there is a wide spread in the incidence among counties that met our climate‐constrained niche model thresholds (Figure 1). Some of this spread may be caused by fine‐scale variations in agriculture, dust storms, health care infrastructure, epidemiological reporting, and other natural and socioeconomical factors known to influence *Coccidioides* spp. abundance and disease incidence (Gorris et al., 2018; Louie et al., 1999; Tong et al., 2017; Williams et al., 1979). Following a similar approach to our endemicity analysis, we used the climate projections from CMIP5 to estimate future changes in potential Valley fever incidence.

### 2.5 Projections of Human Population

To isolate the effects that climate change alone may have on the number of people who contract Valley fever, in our main analysis we assumed an invariant human population in the United States. However, United States population is projected to increase throughout the 21st century (Hauer, 2019), which may expose more people to *Coccidioides* ssp. and lead to more Valley fever cases. To estimate the combined effect of both climate change and increasing population, we used future projections of human population from the shared socioeconomic pathways (SSPs; Hauer, 2019) to calculate future population levels within the Valley fever endemic region. The county‐level human population projections we used take into account age, sex, and race and were specifically tailored for the United States (Hauer, 2019).

The SSPs describe how socioeconomic factors such as population, economic growth, and technological development evolve in the absence of climate change or climate policy (O'Neill et al., 2014). We used both SSP2, a scenario in which there is moderate population growth in the United States throughout the 21st century, and SSP5, a scenario in which there is large population growth (O'Neill et al., 2014). Our 2007 (mean of years 2000–2015) baseline United States population is 300 M (U.S. Census Bureau, 2011, 2016). By 2095, SSP2 projects the total United States population to be 454 M and SSP5 projects it to be 690 M (Hauer, 2019). We examined each SSP population scenario in combination with the RCP4.5 and RCP8.5 climate scenarios.

## 3 Results

### 3.1 Estimating the Current Spatial Extent of Valley Fever Endemicity

**Figure 3:**
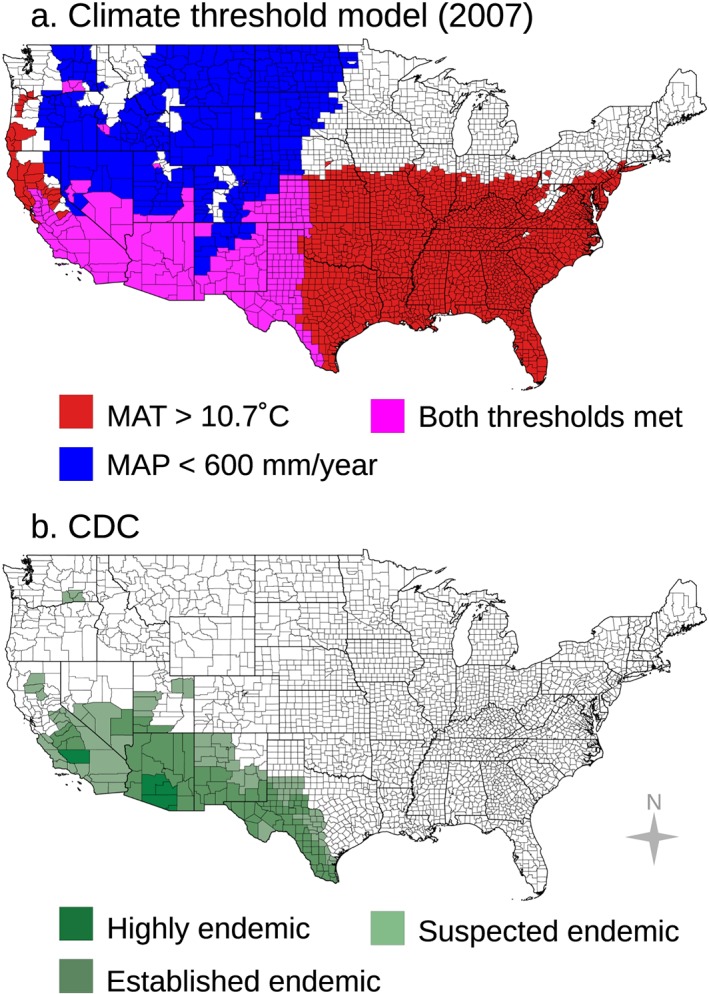
(a) Counties our climate‐constrained niche model identify as endemic (with a mean annual temperature greater than or equal to 10.7°C and a mean annual precipitation level less than or equal to 600 mm/year) are colored in magenta. (b) There is reasonable agreement between this set of counties and the endemic region identified by the CDC. Counties shown in red in panel a have a mean annual temperature greater than or equal to 10.7°C but unsuitable mean annual precipitation (greater than 600 mm/year). Counties shown in blue have a mean annual precipitation level less than or equal to 600 mm/year but unsuitable mean annual temperature (less than 10.7°C). Counties in white our model defines as unsuitable according to both thresholds.

We used our climate‐constrained niche model to map counties potentially endemic to Valley fever for the 2007 baseline period (mean of years 2000–2015; Figure 3a). Counties where mean annual temperature and mean annual precipitation are suitable for Valley fever endemicity are shown in magenta. Counties with suitable temperature but unsuitable precipitation are shown in red. Likewise, counties with suitable precipitation but unsuitable temperature are shown in blue. Counties where both temperature and precipitation are unfavorable are shown in white. This analysis reveals that precipitation limits the area endemic to Valley fever to the north along the coast of the Pacific Northwest and to the east across eastern Texas, Oklahoma, and Kansas, whereas temperature limits the northern range of Valley fever endemicity in many interior western states.

Using our climate niche model, we estimate that 217 counties may currently be endemic to Valley fever. These counties span 12 states—Arizona, California, Colorado, Idaho, Kansas, Nebraska, Nevada, New Mexico, Oklahoma, Texas, Utah, and Washington State. Using the 2007 baseline county population estimate, approximately 47.5 M people live within this endemic region (U.S. Census Bureau, 2016).

The niche model predicts a spatial pattern of endemicity that is broadly similar to the map produced by the CDC but with several notable differences (Figure 3b). Of the 170 counties identified by the CDC as endemic, the niche model classifies 110 counties as potentially endemic. Of the 60 counties not classified as endemic by our model but identified by the CDC, many are located in southwestern Utah, northwestern New Mexico, and southcentral Texas. Compared to the CDC map, our model also omits a few counties that previously experienced localized outbreaks of Valley fever. These outbreaks include cases contracted in Dinosaur National Monument and Duchesne County in Utah (Petersen et al., 2004), where *Coccidioides* spp. is thought to survive in isolated areas with high soil temperatures, and cases associated with archeological sites in northern California in Tehama and Butte Counties (Werner et al., 1972; Werner & Pappagianis, 1973).

Our niche model predicts 107 counties as endemic that the CDC model did not identify as endemic. The niche model predicts that endemic areas extend farther north throughout the Great Plains and Central Valley of California. These areas include several states that are absent from the CDC map, including Colorado, Idaho, Kansas, Nebraska, and Oklahoma. The model identifies the two most populous counties in Idaho–Ada and Canyon County–as potentially endemic, including the city of Boise (U.S. Census Bureau, 2016).

One striking similarity between our model estimate and the CDC map is the identification of endemicity in three counties in southeastern Washington State, originally thought to be well outside the endemic region. These counties were recently added to the CDC map after an outbreak of Valley fever cases was reported from 2010–2011 (Marsden‐Haug et al., 2012). Since then, *C. immits* has been extracted from Washington State soils (Litvintseva et al., 2014).

Considering the CDC endemic map as truth, our model identifies 2,831 counties in the United States as true negatives (TN; nonendemic), 110 counties as true positive (TP; endemic), 107 counties as false positives, and 60 counties as false negatives (FN). This corresponds to a 94.6% accuracy rate ([TP+TN]/total) for predicting endemic counties in the United States (a 5.4% error rate) and a 64.7% recall rate (TP/[TP+FN]).

The Maxent ecological niche models that we ran as a sensitivity analysis produces similar, but more conservative patterns of contemporary endemicity when compared to our climate‐constrained niche model. Both the two‐variable and three‐variable Maxent models have higher accuracy rates (96.3% and 96.8%, respectively; Table S2). However, the two‐variable Maxent model considerably underestimates the number of endemic counties compared to the CDC map, with a 37.6% recall rate; it yields more false negatives (106) and fewer true positives (64) when compared to the climate‐constrained niche model. The relative contributions of the environmental variables in the two‐variable Maxent model are 80% for mean annual precipitation and 20% for mean annual temperature, highlighting the importance of precipitation in structuring contemporary areas of endemicity. The three‐variable Maxent model performs better than the two‐variable Maxent model but again yields more false negatives (79) and fewer true positives (91) compared to the climate‐constrained niche model. The relative contributions of variables in the three‐variable model are 75% for mean annual precipitation, 25% for mean annual January temperature, and less than 0.1% for mean annual July temperature, which again demonstrates the importance of precipitation and suggests that winter temperatures may be more important than summer temperatures in structuring the spatial pattern of endemicity.

Overall, our simple, two‐variable climate‐constrained niche model provides a reasonable regional‐scale depiction of the area endemic to Valley fever. Other factors such as soil characteristics and competition among microorganisms may further refine where *Coccidioides* spp. is present on finer spatial scales. Additionally, *Coccidioides* spp. may be able to adapt to different soil environments (Colson et al., 2017). Recognizing that many additional processes contribute to *Coccidioides* spp. abundance and disease dynamics at finer spatial scales, our model may enable preliminary exploration of climate change impacts on areas affected by Valley fever throughout the 21st century.

### 3.2 Estimating the Future Spatial Extent of Valley Fever Endemic Regions

**Figure 4:**
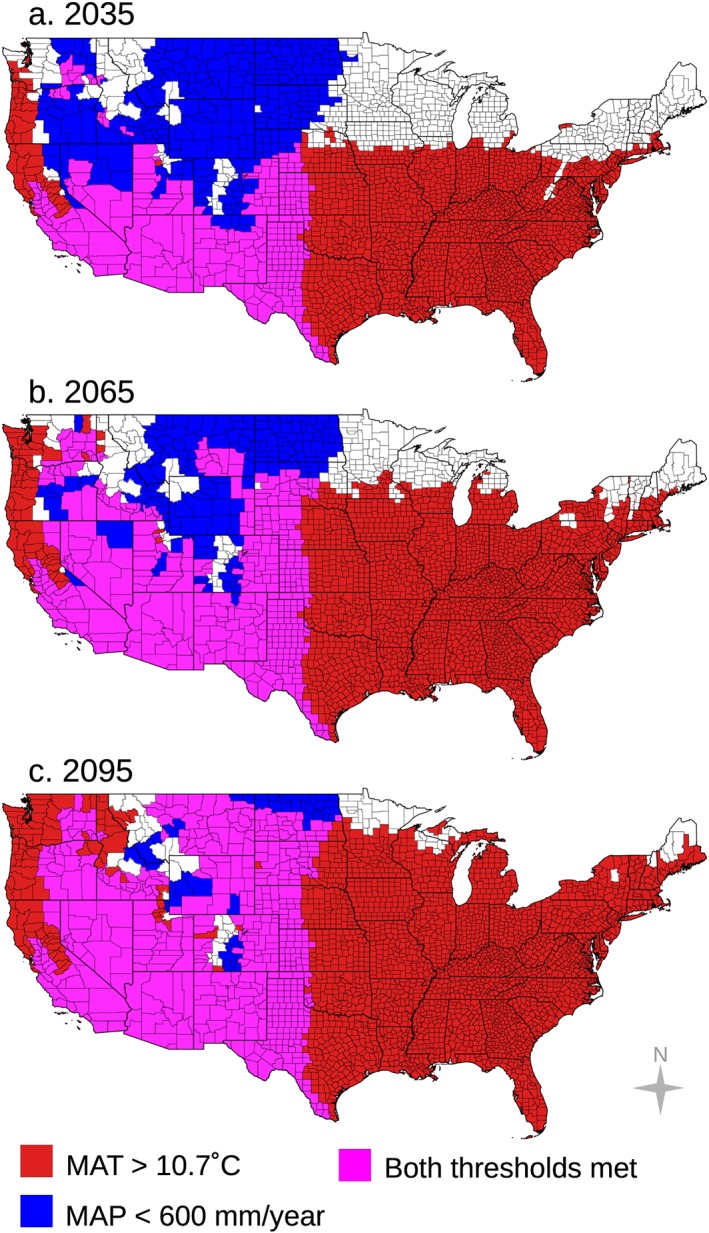
For the representative concentration pathway (RCP) 8.5 climate change scenario, areas where climate will permit Valley fever endemicity are shown for the years (a) 2035, (b) 2065, and (c) 2095. Areas where mean annual temperature will permit endemicity are shown in red, areas where mean annual precipitation will permit endemicity are shown in blue, and areas where both temperature and precipitation will permit endemicity are shown in magenta, following the color scheme used in Figure 3. The area endemic to Valley fever will extend farther north in future decades, especially in the rain shadows of the Sierra Nevada and Rocky Mountain Ranges. Precipitation will play a key role in determining which areas become endemic through time, as greater rainfall and moisture availability will limit the eastward extent of Valley fever as well as its presence in the Pacific Northwest and in western counties at higher elevations.

**Figure 5:**
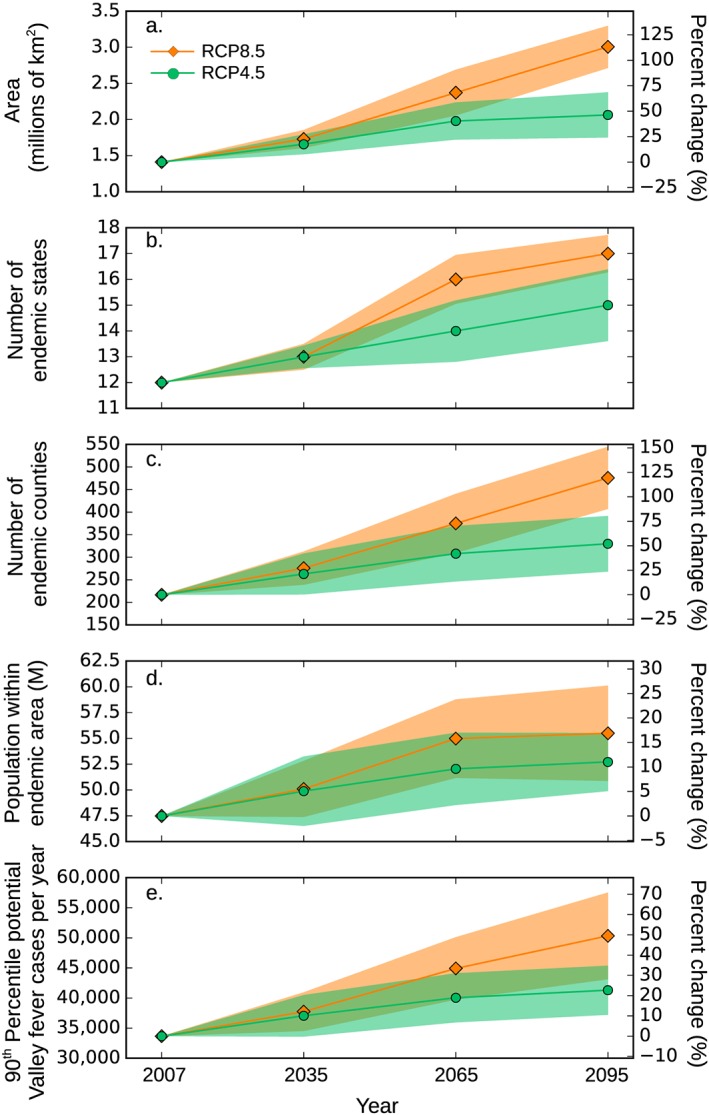
Time series of change in (a) the total area potentially endemic to Valley fever, (b) the number of endemic states, (c) the number of endemic counties, (d) the number of people living within endemic regions, and (e) the estimated number of annual cases from 2007 to 2095 for both representative concentration pathway (RCP) 8.5 and RCP4.5 climate scenarios. The shaded areas are the standard deviation describing variation among the 30 Coupled Model Intercomparison Project Phase 5 (CMIP5) Earth system models used in our analyses.

We applied our climate‐constrained niche model to identify counties that may become endemic to Valley fever in the future for the moderate (RCP4.5) and high (RCP8.5) climate warming scenarios. Over time, the area of climate‐constrained endemicity is predicted to expand northward, most notably throughout the Great Plains and in the rain shadows of the Sierra Nevada and Rocky Mountain Ranges (Figures 4 and S3). For the high climate warming scenario (RCP8.5), the model predicts that by the end of the 21st century, the area endemic to Valley fever will more than double (a 113% increase), the number of states with Valley fever endemicity will increase from 12 to 17, the number of counties with endemicity will increase from 217 to 476, and the number of people living within the endemic region will increase by 17% (Figure 5). The smaller relative change in human exposure compared to endemic area is caused by increases in endemicity in many western counties that have relatively low population (and follows our assumption here of an invariant population). For the moderate climate warming scenario (RCP4.5), the model predicts that by the end of the century the expansion of Valley fever endemic area will be considerably smaller than for the RCP8.5 scenario, increasing by only about 46% (Figures S3 and 5a). Other Valley fever disease metrics also change more slowly for RCP4.5 (Figures 5b–5e). The contrast between the two scenarios highlights the importance of climate change mitigation as a means for limiting the health impacts of Valley fever, especially for more northern states (Table S3).

By 2035 for RCP8.5, we predict the climate‐constrained range of Valley fever will expand into northern Utah and eastern Colorado. By 2065, southern Idaho, Nebraska, southeastern Montana, and South Dakota will become endemic, and by 2095, Valley fever will enter North Dakota and move farther north in Montana. The Valley fever endemic region will expand northward in dry western states primarily as a consequence of warming that pushes mean annual temperatures above the temperature threshold required for disease establishment. From our baseline time period to 2095 for RCP8.5, 242 counties will become endemic to Valley fever because of warming above the temperature threshold, 20 counties will become endemic because of drying below the precipitation threshold, and 3 counties will become unsuitable for endemicity because of increases in precipitation.

Precipitation has a key role in determining whether a county becomes endemic in the future. By 2095 for RCP8.5, most of the western United States will have a climate that permits Valley fever endemicity, except for counties near the central and northern Pacific Coast and counties at higher elevations in mountain ranges. Northern California, western Oregon, and western Washington State will meet the mean annual temperature threshold yet will be shielded from becoming endemic because of high levels of precipitation. The eastward extent of the climate‐constrained endemic range across the Great Plains is also limited for contemporary and future periods by precipitation, with a sustained north‐south barrier occurring near the 100°W meridian. This axis corresponds to a zonal atmospheric water vapor gradient where dry, continental air from the southwestern United States meets moist, warm maritime air from the Gulf of Mexico, creating a sharp increase in moisture availability to the east (Lin, 2007).

**Figure 6:**
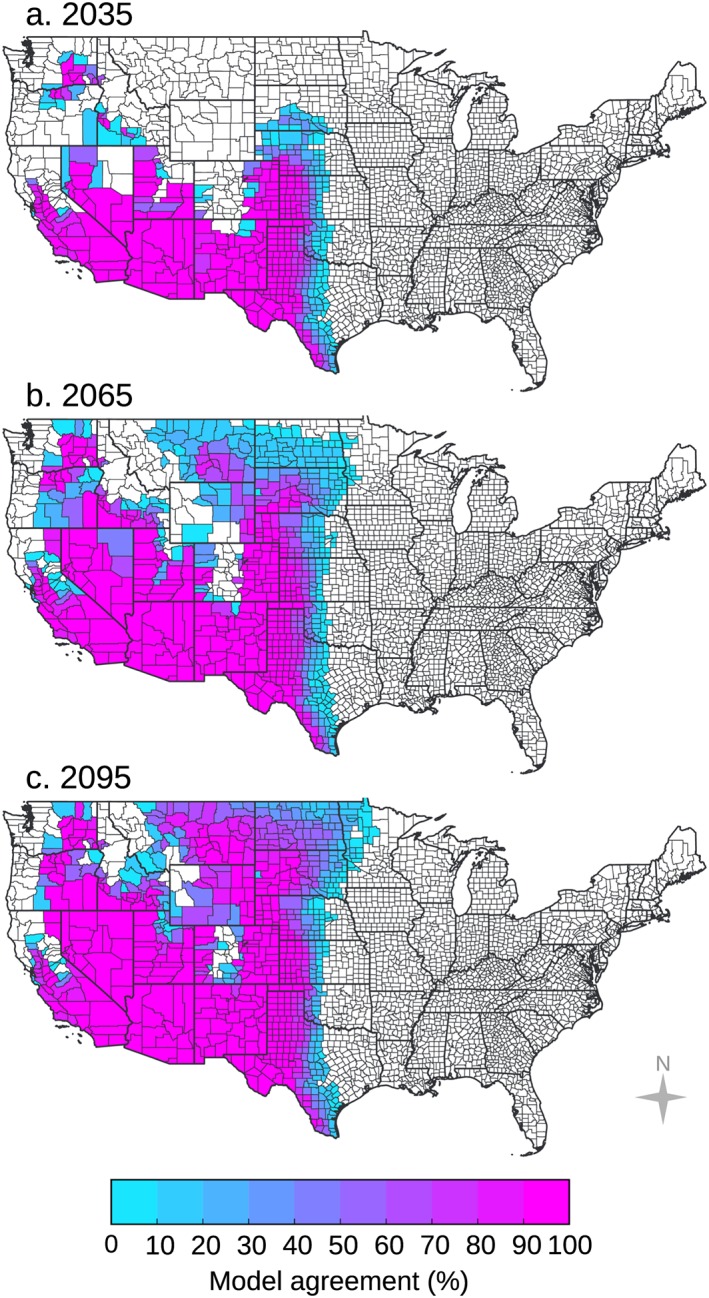
There is strong model agreement throughout the majority of the area we estimate as endemic to Valley fever for the RCP8.5 climate scenario in years (a) 2035, (b) 2065, and (c) 2095. The model agreement shows a measure of uncertainty for the counties along the edge of the endemic area. Some models predict that the endemic range in 2095 will expand into counties as far east as western Minnesota. Percent model agreement was calculated as the number of individual CMIP5 models that predict the county will have a climate that permits endemicity, divided by the total number of models (*n* = 30), as projected by the climate‐constrained niche model.

We calculated the percent of the individual CMIP5 model simulations that are in agreement that each county will have a climate that permits Valley fever endemicity for the RCP4.5 and RCP8.5 scenarios. There is strong model agreement across the majority of the projected endemic region (Figures 6 and S4). By 2095 for RCP8.5, some models predict that Valley fever will be endemic farther east throughout the Central Plains, even into Minnesota. However, there is still a clear climate control on the eastern boundary of endemicity driven by the moisture gradient along the 100°W meridian. There is also strong agreement that several high elevation counties within the Rocky Mountains, as well as counties along the northern Pacific Coast, will remain outside the zone of endemicity.

As a sensitivity analysis, we ran projections of our Maxent ecological niche models for RCP8.5. Both the two‐variable and three‐variable Maxent models also predict an expansion of areas endemic to Valley fever along the leeside of the Rocky Mountains and in the dry inland areas of the Pacific Northwest including southeastern Washington State. By 2095 for RCP8.5, the two‐variable Maxent model identifies that 15 states will have a climate that permits endemicity and the three‐variable Maxent model identifies 14 states (Table S2). We calculated the relative change in the population living within the Valley fever endemic area to compare across models, not considering changes in human population. Our climate‐constrained threshold model predicts that the population living within the Valley fever endemic area will increase by 6% in year 2035, by 16% in 2065, and by 17% in 2095. Similarly, the two‐variable Maxent model predicts a 5% increase in 2035, a 12% increase in 2065, and an 18% increase by 2095. Projections using the three‐variable Maxent model show similar changes and yield a 16% increase in the population living within the Valley fever endemic area by 2095. Although the Maxent models are more conservative in estimating the area endemic to Valley fever for the contemporary period, the projected pattern of Valley fever expansion is broadly consistent across all three models. The three‐variable Maxent model that includes both January and July mean annual temperatures as explanatory variables allows us to better represent biological limits on the fitness of *Coccidioides* spp. to inhabit regions that experience exceptionally cold winters or hot summers. This more complex model still yields a pattern of future expansion that is similar to the simpler models that use mean annual climate variables.

### 3.3 Estimating Current and Future Mean Annual Valley Fever Incidence

**Figure 7:**
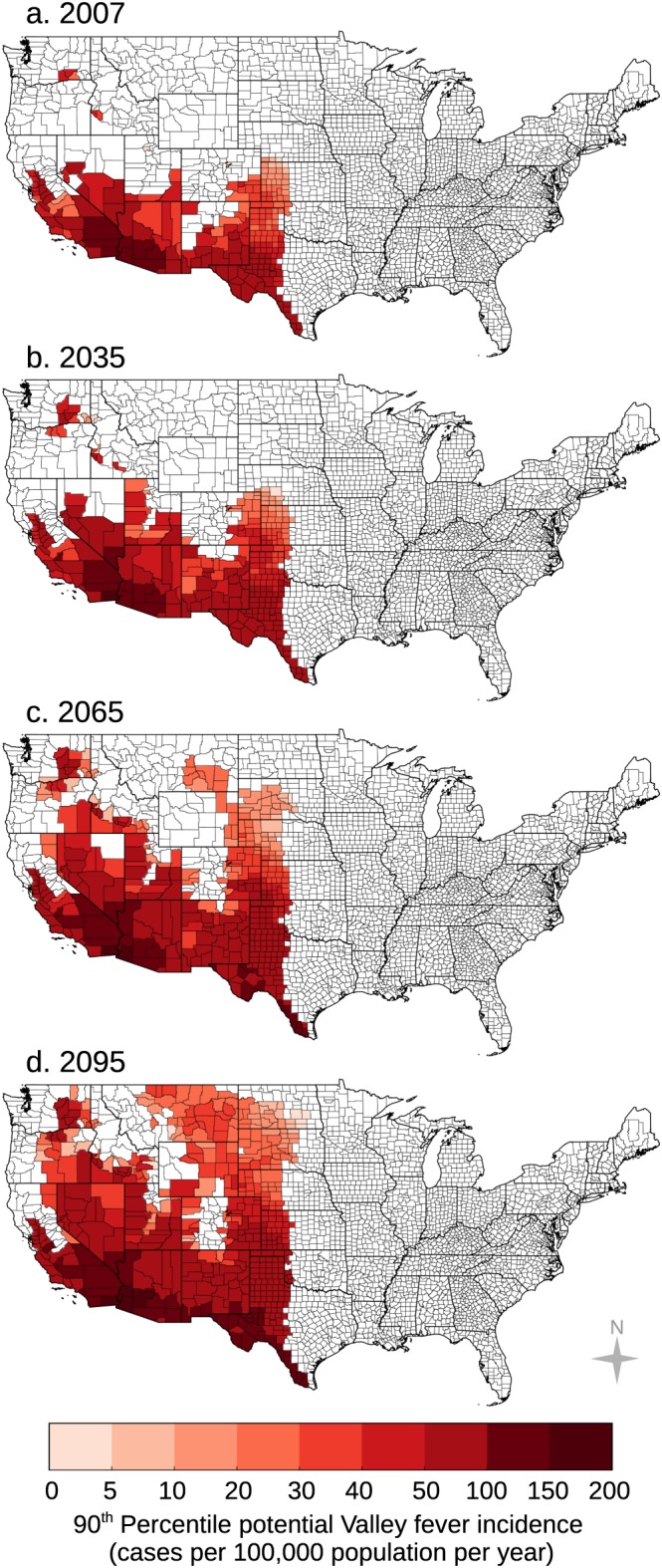
We estimated an upper bound of future Valley fever incidence using a 90th percentile regression model for (a) our 2007 baseline period, (b) 2035, (c) 2065, and (d) 2095 for representative concentration pathway (RCP) 8.5. Over time, our model predicts Valley fever incidence will increase throughout the extreme southwestern US and the southern Great Plains. Incidence will also increase throughout the Central Valley of California and in the northwestern United States.

We estimated an upper bound of Valley fever incidence by performing quantile regression on observed Valley fever incidence and mean annual temperature and precipitation (Figure 7). For our baseline period, our model predicts that mean annual Valley fever incidence is likely to be greatest in the extreme southwestern United States and southwestern Texas (Figure 7). The model also predicts high incidence in the Central Valley of California. For the baseline period, our model predicts up to 34,460 potential cases of Valley fever within Arizona, California, Nevada, New Mexico, and Utah, compared to approximately 9,500 observed cases per year (CDC, 2018a).

We then applied our quantile regression model to future climate projections for both RCP4.5 and RCP8.5. Our model predicts that Valley fever incidence will increase over time throughout the extreme southwestern United States, southern Great Plains, Central Valley of California, and the northwestern United States (Figures 7 and S5). Using our baseline (invariant) human population estimates, we transformed incidence projections into the number of Valley fever cases (Figure 5e). The number of potential cases each year for RCP8.5 is projected to increase by 12% in the year 2035 and by 50% in the year 2095.

### 3.4 Compounding effects of climate change and human population projections on Valley fever

Increasing United States population will compound disease impacts caused by climate change. By 2095 for RCP8.5 assuming an invariant population, we estimate that the number of people living in the Valley fever endemic area will be 55.5 M (Table 1). When we account for both climate change and increasing population, this number increases by 32% (73.2 M) for the SSP2 population scenario and by 44% (80.1 M) for the SSP5 population scenario. In concert, the number of potential Valley fever cases will increase by the same percent. The compounding effect between climate change and increasing population in the dry southwestern United States highlights the importance of developing more effective approaches for measuring and modeling geospatial patterns of *Coccidioides* spp. abundance and disease risk.

**Table 1:** Compounding Effects of Climate Change and Increasing Human Population on the Number of People in Millions Living in the Endemic Region for Valley Fever in the Years 2035, 2065, and 2095, Relative to our 2007 Baseline Population Estimate of 47.5 M

	RCP4.5 Climate			RCP8.5 Climate		
	2035	2065	2095	2035	2065	2095
No change in population (M)	49.9	52.1	52.7	50.1	55.0	55.5
SSP2 population scenario (M)	65.7	68.6	69.7	66.0	72.6	73.2
SSP5 population scenario (M)	71.9	75.0	76.2	72.2	79.4	80.1

Abbreviations: RCP, representative concentration pathway; SSP, shared socioeconomic pathways.

## 4 Discussion

### 4.1 Biogeography of Valley Fever Expansion

Our analysis identifies that a primary pathway for Valley fever expansion lies in the rain shadow of the Rocky Mountains. By the end of the 21st century, the climate‐constrained area endemic to Valley fever will extend from the southern through the northern Great Plains. This is a predominant region for agriculture, which has a positive correlation with Valley fever incidence (Gorris et al., 2018). Further, climate projections indicate that this region will experience an increased risk of drought (Cook et al., 2015). Together, intensifying drought and agriculture may increase the amount of dust loading and thus human exposure to *Coccidioides* spp. It is notable in this context that the Valley fever expansion pathway predicted by our model is through areas affected by the 1930s Dust Bowl (Burnette & Stahle, 2013).

Not all states throughout the Great Plains are required to report Valley fever cases, which may limit our ability to monitor the potential spread of this disease. States in the Great Plains that do report have had minimal cases in recent years (CDC, 2019). There is plausible evidence, however, that *Coccidioides* spp. inhabited this region before. Two buffalo that were radiocarbon dated to be 8500 years old, collected near Milburn, Custer County, Nebraska, showed signs of a fungal infection consistent with Valley fever; the buffalo may have migrated through endemic regions in the south before meeting their demise in Nebraska, or alternatively, the central Great Plains was an endemic region in the past (Morrow, 2006).

### 4.2 Increasing Costs of Valley Fever for Human Health

We expect the total number of Valley fever cases and subsequently total cost of disease will increase in concert with the expanding endemic area. Roughly 45% of people with Valley fever are hospitalized (Sondermeyer et al., 2013; Tsang et al., 2010). The estimated median total hospital charge per person in California from 2000–2011 was $55,000 (assuming 2011 USD; Sondermeyer et al., 2013). Based upon this hospitalization rate, the median total hospital charges (about $58,000 in 2015 USD), and the number of observed cases from 2000 to 2015 (149,286 cases), we estimate that total hospitalization costs are about $244 M per year (2015 USD) for our baseline period. Based on our predicted changes in the relative number of Valley fever cases (and assuming no change in human population), we estimate that hospitalization costs due to climate change alone for the RCP8.5 scenario will rise to $274 M per year in 2035, $326 M per year in 2065, and $365 M per year in 2095 (2015 USD). These estimates do not include other costs associated with outpatient care and medications, missed days of work, or childcare (Colby & Ortman, 2014; Sondermeyer et al., 2013; Tsang et al., 2010), nor do they account for the compounding effects of future changes in United States population described above.

### 4.3 Improving Future Projections and Sources of Uncertainty

Our derived maps of Valley fever endemicity in 2035, 2065, and 2095 describe the disease range constrained solely by future climate. For these areas to become endemic, however, *Coccidioides* spp. needs to physically move into these new areas. This migration may be accomplished by the atmospheric transport of fungal spores in dust or by migration of infected animals, such as rodents (Taylor & Barker, 2019). To reduce uncertainties regarding rates of spread, more work is needed to systematically map the presence of *Coccidioides* spp. in both soils and atmospheric dust throughout the western United States.

Our map of the area currently endemic to Valley fever may be helpful in the design of future sampling campaigns to gather occurrence data of *Coccidioides* spp. Once the presence of *Coccidioides* spp. in soils has been systematically mapped, we will be able to build a spatially explicit environmental niche model for *Coccidioides* ssp. directly from environmental surveillance data instead of epidemiological case reports (Miller, 2010; Peterson, 2006) and use this model to determine the response of the fungi to climate change (e.g., Escobar et al., 2016; Romero‐Alvarez et al., 2017). As more positive occurrences of *Coccidioides* spp. in the soil are obtained, it will become increasingly critical to simultaneously measure soil properties such as alkalinity, pH, salinity, soil type, soil texture, along with the diversity and presence of other soil microbes to further refine the environmental controls on fungal presence and abundance. High‐resolution occurrence maps could also help disentangle controls on disease incidence arising from different *Coccidioides* species (Baptista‐Rosas et al., 2007; Colson et al., 2017; Lauer, 2017) as well as the impacts of heterogeneity in elevation and climate conditions within each county, especially for large counties throughout the western United States that span mountainous areas.

Concurrently, improved monitoring and reporting of Valley fever cases in states that currently have low or marginal disease incidence would allow for a more accurate delineation of contemporary climate controls. This is most critical for states where current climate conditions permit endemicity (Figure 3), yet the state is not currently reporting, including Colorado, Idaho, Kansas, Oklahoma, and Texas (CDC, 2018a). Proactive surveillance in states where climate does not currently permit endemicity but may in the future will help with monitoring disease spread.

Another factor that will likely modulate the number of Valley fever cases in the future is changes in the seasonal and interannual variability of precipitation. Precipitation in California is projected to shift to more intense periods of heavy and extreme rainfall, with moderate to small changes in the overall amount (Polade et al., 2017; Swain et al., 2018). These periods of greater moisture availability may increase fungal growth, while longer and more intense dry periods may enhance dust production and dispersal. In Arizona, summer rainfall brought by the North American monsoon is projected to weaken (Pascale et al., 2017), potentially leading to drier and dustier summers. It is also important to recognize that there is significant low‐frequency (decadal) internal variability in precipitation in the western United States, driven, for example, by the Pacific Decadal Oscillation, that may seemingly dampen or amplify the effects of climate change (e.g., Lehner et al., 2018). In our analysis, variability in precipitation causes some counties to switch back and forth over time in terms of their designation as endemic. For example, the estimated number of California counties endemic to Valley fever for RCP8.5 increases from 28 counties in 2035 to 31 counties in 2065, but then decreases to 30 counties in 2095 due to an increase in precipitation in San Francisco County, which was considered endemic in 2065. Evidence of precipitation variability can also be seen in the maps of precipitation change for RCP4.5 (Figure S2), where many areas that are drier in 2035 become wetter again in 2065, contrary to the stronger unidirectional pattern of change associated with anthropogenic forcing in RCP8.5.

We used a large set of CMIP5 model simulations to calculate the average projections of climate change for the United States. Although some models perform better than others for the United States compared to historical observations, the multimodel mean tends to provide a reliable estimate of contemporary surface climate (Sheffield et al., 2013). With improved representation of ocean and atmospheric dynamics and higher spatial resolution, simulations contributed to the 6th Coupled Model Intercomparison Project (CMIP6; Eyring et al., 2016) will likely reduce uncertainties in future projections of temperature and precipitation for the United States (Stouffer et al., 2017). The higher quality climate information, along with improved downscaling techniques, will provide better boundary conditions for statistical and mechanistic models predicting changes in Valley fever endemic regions. However, uncertainty in climate projections is only one of the several different types of uncertainty limiting our ability to predict Valley fever endemicity.

Our model draws upon Valley fever incidence data, which implicitly links *Coccidioides* spp. presence with human cases of Valley fever. An important next step is the development of a mechanistic model, which separately simulates *Coccidioides* spp. abundance, transmission efficiency, and host heterogeneity as a function of different environmental and human demographic variables. As research on Valley fever and *Coccidioides* spp. continues, additional information such as the possible role of mammals in the fungal life cycle (Barker, 2018; Taylor & Barker, 2019), variations in ecological traits and ecosystems linked to different species of *Coccidioides* (Barker et al., 2012; Colson et al., 2017), and microbial competition (Lauer et al., 2019) will need further consideration for integration into both mechanistic and statistical models of disease incidence. This will be especially important if we learn that different *Coccidioides* species have different virulence and tolerances for environmental controls, as this could affect the dispersal of disease and health impacts caused by climate change. As more occurrences of *Coccidioides* spp. in the soil are documented, adding any soil characteristics that limit the presence of the fungi into the model, such as alkalinity, salinity, soil type, and soil texture, may further refine the endemic area (Baptista‐Rosas et al., 2007; Colson et al., 2017; Fisher et al., 2007; Maddy, 1957).

### 4.4 Coccidioidomycosis in a Global Context

Disease surveillance efforts throughout the United States and the comprehensive Valley fever case data set provided the foundation for our study. However, Valley fever is not limited to the United States. Our model, as well as the CDC endemicity model, depicts Valley fever endemicity spanning the United States‐Mexico border. It is well known that *Coccidioides* spp. is present in Mexico; however, there has been minimal disease surveillance within the country (CDC, 2018b; Laniado‐Laborin, 2007). Our future projections indicate that the climate‐constrained endemic region may also extend north to the United States‐Canada border by the end of the 21st century, potentially introducing *Coccidioides* spp. to a new country.

We found that the area endemic to Valley fever in the United States, as well as the number of cases per year, will increase in response to climate change. Patterns of future change may be similar in other endemic areas within Central and South America. Apart from Mexico, countries that are likely endemic to Valley fever include Guatemala, Honduras, Argentina, Brazil, Paraguay, Bolivia, Venezuela, and Columbia (Colombo et al., 2011; Laniado‐Laborin, 2007). International collaboration and Valley fever surveillance in these regions will help delineate the endemic boundaries, provide further information regarding the environmental factors structuring disease presence and incidence, and increase physician awareness (Cat et al., 2019).

### 4.5 Importance of Integrating Valley Fever Into Future Climate Change Assessments

The U.S. Global Change Research Program recently suggested that climate change may alter the spatial extent and number of Valley fever cases (Crimmins et al., 2016). Our study provides a first estimate to quantitatively describe this change. Furthermore, the Fourth National Climate Assessment report for the United States recognized the implications of drought on the interannual variability of cases (Ebi et al., 2018). Although the area currently endemic to Valley fever is relatively smaller than other infectious diseases, like West Nile Virus (CDC, 2018c), we expect that there may be similar or even larger negative health impacts from the exposure of new communities to Valley fever in response to climate change. In fact, recent mortality rates from Valley fever are similar, if not larger, than those reported for West Nile virus. There are approximately 110 deaths per year from West Nile virus in the United States (mean 1999–2016; CDC, 2018c) compared to approximately 200 deaths per year from Valley fever (mean 1990–2008; CDC, 2018a). Further, Valley fever cases have increased considerably since 2008, suggesting that there may be additional negative impacts from this disease.

## 5 Conclusions

We combined a multistate database of VFI observations and climate projections to predict how climate change may influence the endemic area and number of Valley fever cases in the United States. Using our climate‐constrained niche model, we found that the endemic area to Valley fever, as well as the number of cases per year, will increase in response to climate change. As temperatures increase and precipitation patterns change, most of the western United States will meet climate thresholds necessary for Valley fever endemicity. Through time, we found that the endemic area will expand northward, most notably through the Great Plains. Expansion of the endemic area is suppressed farther east by regional increases in precipitation and the presence of moist air from the Gulf of Mexico. By 2095 for a high climate warming scenario (RCP8.5), our model predicts that 476 counties across 17 states will have a climate that permits Valley fever endemicity. This could result in up to 50% more annual Valley fever cases, before taking into account the compounding effect of future increases in human population. Estimating the regions that may become endemic to Valley fever can mitigate the health effects of this disease, as it will allow health care providers and citizens to prepare in advance. Our research is an example of the necessary bridge between climate science and human health as climate change reshapes areas endemic to infectious diseases.

## Conflict of Interest

The authors declare no conflicts of interest relevant to this study.

## Supporting information



Supporting Information S1
