# Supplementary material for: Expansion of Coccidioidomycosis Endemic Regions in the United States in Response to Climate Change
Source: Geohealth. 2019 Oct 10;3(10):308–27. doi: 10.1029/2019GH000209 (PMC7007157; doi:10.1029/2019GH000209)
Supplement: Supplementary file 1 — Supporting Information S1 [file GH2-3-308-s001.docx]

***Supporting Information:***

**Expansion of coccidioidomycosis endemic regions in the United States in response to climate change**

Morgan E. Gorris^1^, Kathleen K. Treseder^2^, Charles S. Zender^1^, James T. Randerson^1^

^1^Department of Earth System Science, University of California, Irvine, California, USA

^2^Department of Ecology and Evolutionary Biology, University of California, Irvine, California, USA

**Table S1.** BCSD models used for climate projections, averaged to the county-level from 0.125° × 0.125° resolution.

| **Modeling Center** | **Institute ID** | **Model Name** | **Num. endemic counties 2095 RCP4.5** | **Num. endemic counties 2095 RCP8.5** | **Citation** |
| --- | --- | --- | --- | --- | --- |
| Commonwealth Scientific and Industrial Research  Organization (CSIRO) and Bureau of Meteorology  (BOM), Australia | CSIRO-BOM | ACCESS1.0 | 376 | 537 | Collier, M. and Uhe, P. (2012). CMIP5 datasets from the ACCESS1.0 and ACCESS1.3 coupled climate models (CAWCR Technical Report No. 059). Centre for Australian weather and Climate Research. |
| Beijing Climate Center, China Meteorological  Administration | BCC | BCC-CSM1.1  BCC-CSM1.1(m) | 300  431 | 466  456 | Gao F., Xin X., Wu T. (2012). Study on the prediction of regional and global temperature in decadal time scale with BCC_CSM1.1 (in Chinese). *Chinese Journal of Atmospheric Sciences,* 36(6), 1165-1179. |
| Canadian Centre for Climate Modelling and Analysis | CCCMA | CanESM2 | 315 | 343 | Arora, V. K., Boer, G. J., Christian, J. R., Curry, C. L., Denman, K. L., Zahariev, K., et al. (2009). The effect of terrestrial photosynthesis down regulation on the twentieth-century carbon budget simulated with the CCCma Earth System Model. *Journal of Climate*, 22(22), 6066-6088. https://doi.org/10.1175/2009JCLI3037.1 |
| National Center for Atmospheric Research | NCAR | CCSM4 | 293 | 427 | Meehl, G. A., Washington, W .M., Arblaster, J. M., Hu, A., Teng, H., Tebaldi, C., et al. (2012). Climate system response to external forcings and climate change projections in CCSM4. *Journal of Climate*, 25(11), 3661-3683. https://doi.org/10.1175/JCLI-D-11-00240.1 |
| Community Earth System Model Contributors | NSF-DOE-NCAR | CESM1(BGC)  CESM1(CAM5) | 309  337 | 493  506 | Long, M. C., Lindsay, K., Peacock, S., Moore, J. K. and Doney, S. C. (2013). Twentieth-century oceanic carbon uptake and storage in CESM1 (BGC). *Journal of Climate*, 26(18), 6775-6800. https://doi.org/10.1175/JCLI-D-12-00184.1  Meehl, G. A., Washington, W. M., Arblaster, J. M., Hu, A., Teng, H., Kay, J. E., et al. (2013). Climate change projections in CESM1 (CAM5) compared to CCSM4. *Journal of Climate*, 26(17), 6287-6308. https://doi.org/10.1175/JCLI-D-12-00572.1 |
| Centro Euro-Mediterraneo per I Cambiamenti  Climatici | CMCC | CMCC-CM | 398 | 495 | Fogli, P. G., Manzini, E., Vichi, M., Alessandri, A., Patara, L., Gualdi, S., et al. (2009). INGV-CMCC Carbon (ICC): A Carbon Cycle Earth System Model. *CMCC Research Papers*, RP0061, 31. https://doi.org/10.2139.ssrn.1517282  Vichi, M. Manzini, E., Fogli, P., Alessandri, A., Patara, L., Scoccimarro, E., et al. (2011). Global and regional ocean carbon uptake and climate change: sensitivity to a substantial mitigation scenario. *Climate Dynamics*, 37(9-10), 1929-1947. https://doi.org/10.1007/s00382-011-1079-0 |
| Centre National de Recherches Météorologiques /  Centre Européen de Recherche et Formation  Avancée en Calcul Scientifique | CNRM-CERFACS | CNRM-CM5 | 295 | 435 | Voldoire, A., Sanchez-Gomez, E., Mélia, D. S., Decharme, B., Cassou, C., Sénési, S., et al. (2013). The CNRM-CM5. 1 global climate model: description and basic evaluation. *Climate Dynamics*, 40(9-10), 2091-2121. https://doi.org/10.1007/s00382-011-1259-y |
| Commonwealth Scientific and Industrial Research  Organization in collaboration with Queensland  Climate Change Centre of Excellence | CSIRO-QCCCE | CSIRO-Mk3.6.0 | 323 | 462 | Collier, M. A., Jeffrey, S. J., Rotstayn, L. D., Wong, K. K., Dravitzki, S. M., Moseneder, C., et al. (2011). The CSIRO-Mk3. 6.0 Atmosphere-Ocean GCM: participation in CMIP5 and data publication. In International Congress on Modelling and Simulation–MODSIM. *19th International Congress on Modelling and Simulation, Perth, Australia.* |
| LASG, Institute of Atmospheric Physics, Chinese  Academy of Sciences and CESS,Tsinghua  University | LASG-CESS | FGOALS-g2 | 348 | 521 | Li, L., Lin, P., Yu, Y., Wang, B., Zhou, T., Liu, L., et al. (2013). The flexible global ocean-atmosphere-land system model, Grid-point Version 2: FGOALS-g2. *Advances in Atmospheric Sciences*, 30(3), 543-560. https://doi.org/10.1007/s00376-012-2140-6 |
| The First Institute of Oceanography, SOA, China | FIO | FIO-ESM | 254 | 351 | Qiao, F., Song, Z., Bao, Y., Song, Y., Shu, Q., Huang, C. and Zhao, W. (2013). Development and evaluation of an Earth System Model with surface gravity waves. *Journal of Geophysical Research: Oceans*, 118(9), 4514-4524. https://doi.org/10.1002/jgrc.20327 |
| NOAA Geophysical Fluid Dynamics Laboratory | NOAA GFDL | GFDL-CM3  GFDL-ESM2G  GFDL-ESM2M | 314  282  295 | 408  408  438 | Griffies, S. M., Winton, M., Donner, L. J., Horowitz, L. W., Downes, S. M., Farneti, R., et al. (2011). The GFDL CM3 coupled climate model: characteristics of the ocean and sea ice simulations. *Journal of Climate*, 24(13), 3520-3544. https://doi.org/10.1175/2011JCLI3964.1  Dunne, J. P., John, J. G., Adcroft, A. J., Griffies, S. M., Hallberg, R. W., Shevliakova, E., et al. (2012). GFDL’s ESM2 global coupled climate–carbon earth system models. Part I: Physical formulation and baseline simulation characteristics. *Journal of Climate*, 25(19), 6646-6665. https://doi.org/10.1175/JCLI-D-11-00560.1  Dunne, J. P., John, J. G., Shevliakova, E., Stouffer, R. J., Krasting, J. P., Malyshev, S. L., et al. (2013). GFDL’s ESM2 global coupled climate–carbon earth system models. Part II: carbon system formulation and baseline simulation characteristics. *Journal of Climate*, 26(7), 2247-2267. https://doi.org/10.1175/JCLI-D-12-00150.1 |
| NASA Goddard Institute for Space Studies | NASA GISS | GISS-E2-R | 223 | 399 | Schmidt, G. A., Kelley, M., Nazarenko, L., Ruedy, R., Russell, G. L., Aleinov, I., et al. (2014). Configuration and assessment of the GISS ModelE2 contributions to the CMIP5 archive. *Journal of Advances in Modeling Earth Systems*, 6(1), 141-184. https://doi.org/10.1002/2013MS000265 |
| National Institute of Meteorological Research/Korea  Meteorological Administration | NIMR/KMA | HadGEM2-AO | 450 | 552 | Baek, H. J., Lee, J., Lee, H. S., Hyun, Y. K., Cho, C., Kwon, W. T., et al. (2013). Climate change in the 21st century simulated by HadGEM2-AO under representative concentration pathways. Asia-Pacific *Journal of Atmospheric Sciences*, 49(5), 603-618. https://doi.org/10.1007/s13143-013-0053-7  Collins, W. J., Bellouin, N., Doutriaux-Boucher, M., Gedney, N., Hinton, T., Jones, C. D., et al. (2008). Evaluation of the HadGEM2 model. Hadley Center. Technical Note 74. |
| Met Office Hadley Centre (additional HadGEM2-ES  realizations contributed by Instituto Nacional de  Pesquisas Espaciais) | MOHC (additional realizations by INPE) | HadGEM2-CC  HadGEM2-ES | 430  415 | 505  464 | Bellouin, N., Collins, W. J., Culverwell, I. D., Halloran, P. R., Hardiman, S. C., Hinton, T. J., et al. (2011). The HadGEM2 family of met office unified model climate configurations. *Geoscientific Model Development*, 4(3), 723-757. https://doi.org/10.5194/gmd-4-723-2011  Collins, W. J., Bellouin, N., Doutriaux-Boucher, M., Gedney, N., Hinton, T., Jones, C. D., et al. (2008). Evaluation of the HadGEM2 model. Hadley Center. Technical Note 74. |
| Institute for Numerical Mathematics | INM | INM-CM4 | 260 | 424 | Volodin, E. M., Dianskii, N. A. and Gusev, A. V. (2010). Simulating present-day climate with the INMCM4.0 coupled model of the atmospheric and oceanic general circulations. Izvestiya, *Atmospheric and Oceanic Physics*, 46(4), 414-431. https://doi.org/10.1134/S000143381004002X |
| Institut Pierre-Simon Laplace | IPSL | IPSL-CM5A-LR  IPSL-CM5A-MR  IPSL-CM5B-LR | 378  372  290 | 528  660  372 | Dufresne, J. L., Foujols, M. A., Denvil, S., Caubel, A., Marti, O., Aumont, O., et al. (2013). Climate change projections using the IPSL-CM5 Earth System Model: from CMIP3 to CMIP5. *Climate Dynamics*, 40(9-10), 2123-2165. https://doi.org/10.1007/s00382-012-1636-1 |
| Atmosphere and Ocean Research Institute (The  University of Tokyo), National Institute for  Environmental Studies, and Japan Agency for  Marine-Earth Science and Technology | MIROC | MIROC5 | 382 | 568 | Watanabe, M., Suzuki, T., O’ishi, R., Komuro, Y., Watanabe, S., Emori, S., et al. (2010). Improved climate simulation by MIROC5: Mean states, variability, and climate sensitivity. *Journal of Climate*, 23(23), 6312-6335. https://doi.org/10.1175/2010JCLI3679.1 |
| Japan Agency for Marine-Earth Science and  Technology, Atmosphere and Ocean Research  Institute (The University of Tokyo), and National  Institute for Environmental Studies | MIROC | MIROC-ESM  MIROC-ESM-CHEM | 332  414 | 464  512 | Watanabe, S., Hajima, T., Sudo, K., Nagashima, T., Takemura, T., Okajima, H., et al. (2011). MIROC-ESM 2010: Model description and basic results of CMIP5-20c3m experiments. *Geoscientific Model Development*, 4(4), 845-872. https://doi.org/10.5194/gmd-4-845-2011 |
| Max-Planck-Institut für Meteorologie (Max Planck  Institute for Meteorology) | MPI-M | MPI-ESM-LR  MPI-ESM-MR | 299  372 | 403  445 | Giorgetta, M. A., Jungclaus, J., Reick, C. H., Legutke, S., Bader, J., Böttinger, M., et al. (2013). Climate and carbon cycle changes from 1850 to 2100 in MPI‐ESM simulations for the Coupled Model Intercomparison Project phase 5. *Journal of Advances in Modeling Earth Systems*, 5(3), 572-597. https://doi.org/10.1002/jame.20038 |
| Meteorological Research Institute | MRI | MRI-CGCM3 | 221 | 370 | Yukimoto, S., Adachi, Y., Hosaka, M., Sakami, T., Yoshimura, H., Hirabara, M., et al. (2012). A new global climate model of the Meteorological Research Institute: MRI-CGCM3—model description and basic performance. *Journal of the Meteorological Society of Japan*. Ser. II, 90, 23-64. https://doi.org/10.2151/jmsj.2012-A02 |
| Norwegian Climate Centre | NCC | NorESM1-M | 416 | 505 | Bentsen, M., Bethke, I., Debernard, J.B., Iversen, T., Kirkevåg, A., Seland, Ø., et al. (2013). The Norwegian earth system model, NorESM1-M—Part 1: Description and basic evaluation of the physical climate. *Geoscientific Model Development*, 6(3), 687-720. https://doi.org/10.5194/gmd-6-687-2013 |
| **Multi-model mean ± standard deviation** | | | **330 ± 62** | **476 ± 69** |  |

**Table S2.** Model performance metrics relative to the CDC endemicity map and projections of Valley fever metrics for year 2095 for RCP8.5 climate change scenario, for both the climate-constrained niche model and the two Maxent models used in the sensitivity analysis. In the final column, a state is reported as endemic if one or more counties within the state has a climate that permits endemicity.

| **Model** | **Accuracy** | **Recall** | **Percent change in population living in endemic region relative to 2007** | **Number of endemic states in 2095** | **States endemic in 2095** |
| --- | --- | --- | --- | --- | --- |
| **Climate-constrained niche model** | 94.6% | 64.7% | 17% | 17 | AZ, CA, CO, ID, KS, MT, ND, NE, NV, NM, OK, OR, SD, TX, UT, WA, WY |
| **Two-variable Maxent:** annual temperature and annual precipitation | 96.3% | 37.6% | 18% | 15 | AZ, CA, CO, ID, KS, MT, NE, NV, NM, OK, OR, TX, UT, WA, WY |
| **Three-variable Maxent:** January temperature, July temperature, and annual precipitation | 96.8% | 53.5% | 16% | 14 | AZ, CA, CO, ID, KS, NE, NV, NM, OK, OR, TX, UT, WA, WY |

**Table S3.** The number of counties within each state considered endemic for each time period by the climate-constrained niche model for the RCP8.5 climate change scenario. The number of endemic counties for the RCP4.5 climate change scenario is listed second in parenthesis. The total number of counties that are predicted to be endemic within each state during the baseline period is listed in the first column for 2007.

| **State** |  | **2007** |  | **2035** | |  | **2065** | |  | **2095** | |
| --- | --- | --- | --- | --- | --- | --- | --- | --- | --- | --- | --- |
| **Arizona** |  | 15 |  | 15 | (15) |  | 15 | (15) |  | 15 | (15) |
| **California** |  | 29 |  | 28 | (28) |  | 31 | (28) |  | 30 | (28) |
| **Colorado** |  | 11 |  | 22 | (21) |  | 32 | (27) |  | 38 | (30) |
| **Idaho** |  | 2 |  | 7 | (4) |  | 19 | (10) |  | 23 | (13) |
| **Kansas** |  | 31 |  | 32 | (32) |  | 30 | (30) |  | 32 | (32) |
| **Montana** |  | 0 |  | 0 | (0) |  | 8 | (0) |  | 34 | (0) |
| **Nebraska** |  | 3 |  | 12 | (10) |  | 26 | (22) |  | 27 | (26) |
| **Nevada** |  | 6 |  | 9 | (9) |  | 16 | (14) |  | 17 | (14) |
| **New Mexico** |  | 23 |  | 29 | (29) |  | 32 | (31) |  | 33 | (31) |
| **North Dakota** |  | 0 |  | 0 | (0) |  | 0 | (0) |  | 25 | (0) |
| **Oklahoma** |  | 5 |  | 6 | (6) |  | 6 | (5) |  | 9 | (6) |
| **Oregon** |  | 0 |  | 3 | (2) |  | 9 | (6) |  | 14 | (8) |
| **South Dakota** |  | 0 |  | 0 | (0) |  | 22 | (9) |  | 30 | (12) |
| **Texas** |  | 84 |  | 92 | (91) |  | 94 | (87) |  | 99 | (88) |
| **Utah** |  | 5 |  | 14 | (10) |  | 17 | (14) |  | 21 | (14) |
| **Washington** |  | 3 |  | 7 | (6) |  | 12 | (10) |  | 12 | (11) |
| **Wyoming** |  | 0 |  | 0 | (0) |  | 6 | (0) |  | 17 | (2) |

***Supporting Figures***

**
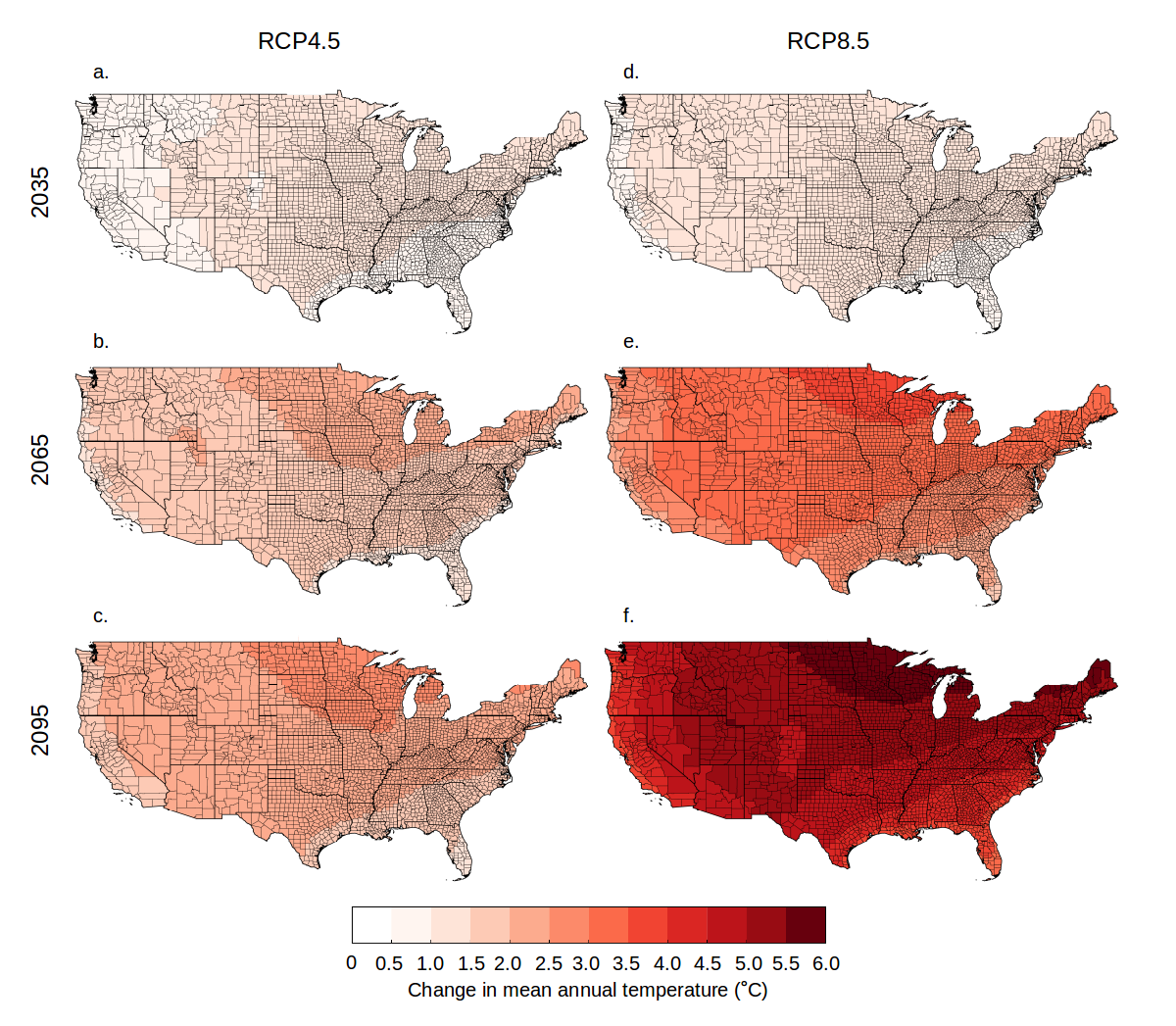
**

**Figure S1.** Mean annual temperature anomalies calculated from the mean of the 30 CMIP5 models under both RCP4.5 (a-c) and RCP8.5 climate scenarios (d-f) in years (a,d) 2035, (b,e) 2065, and (c,f) 2095. Future warming throughout the contiguous US is highest in the northern states and warming is most pronounced for the RCP8.5 climate scenario. These anomalies were estimated relative to a 2000–2015 baseline period (mean of 2007) described in the main text.

**
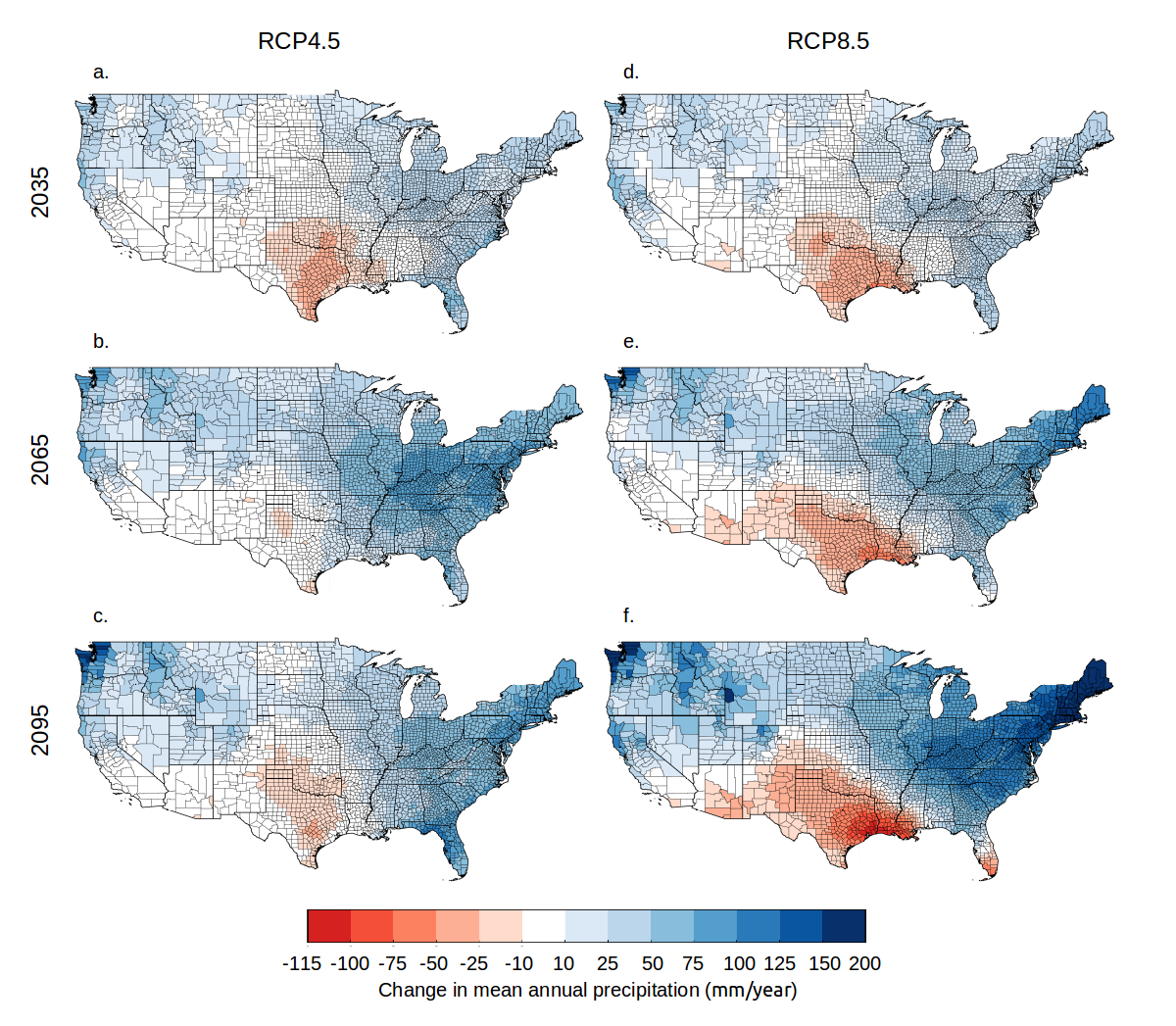
**

**Figure S2.** Mean annual precipitation anomalies calculated from the mean of the 30 CMIP5 Earth system models for both RCP4.5 (a-c) and RCP8.5 climate scenarios (d-f) in years (a,d) 2035, (b,e) 2065, and (c,f) 2095. The south-central Great Plains and southwestern US become drier while the Pacific Northwest and eastern US become wetter. These changes are more pronounced for RCP8.5 climate. These anomalies were estimated relative to a 2000–2015 baseline period (mean of 2007) described in the main text.

**
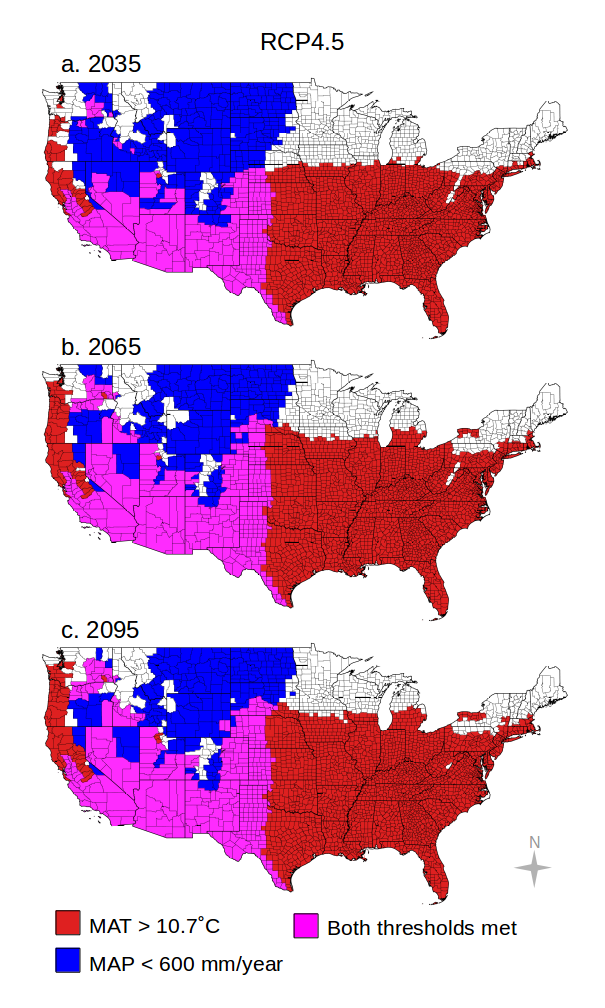
**

**Figure S3.** For the RCP4.5 climate change scenario, areas where climate permits Valley fever endemicity are shown for years (a) 2035, (b) 2065, and (c) 2095. Areas where mean annual temperature permits endemicity are shown in red, areas where mean annual precipitation permits endemicity are shown in blue, and areas where both temperature and precipitation permit endemicity are shown in magenta, following the color scheme used in Figure 3 in the main text. The area endemic to Valley fever will extend farther north in future decades for the RCP4.5 climate scenario, especially in the rain shadows of the Sierra Nevada and Rocky Mountains Ranges.

**
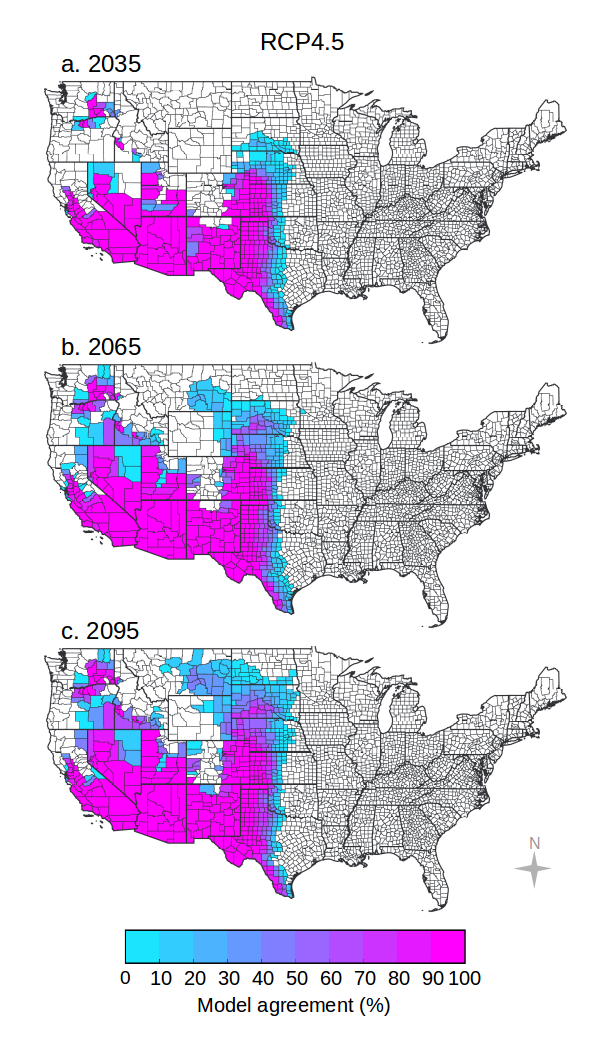
**

**Figure S4.** There is strong model agreement throughout the majority of the area we estimate as endemic to Valley fever for the RCP4.5 climate scenario in years (a) 2035, (b) 2065, and (c) 2095. The model agreement shows a measure of uncertainty for the counties along the edge of the endemic area. Percent model agreement is calculated as the number of individual CMIP5 models that predict the county will have a climate that permits endemicity, divided by the total number of models (n = 30), as projected by the climate-constrained niche model.

**
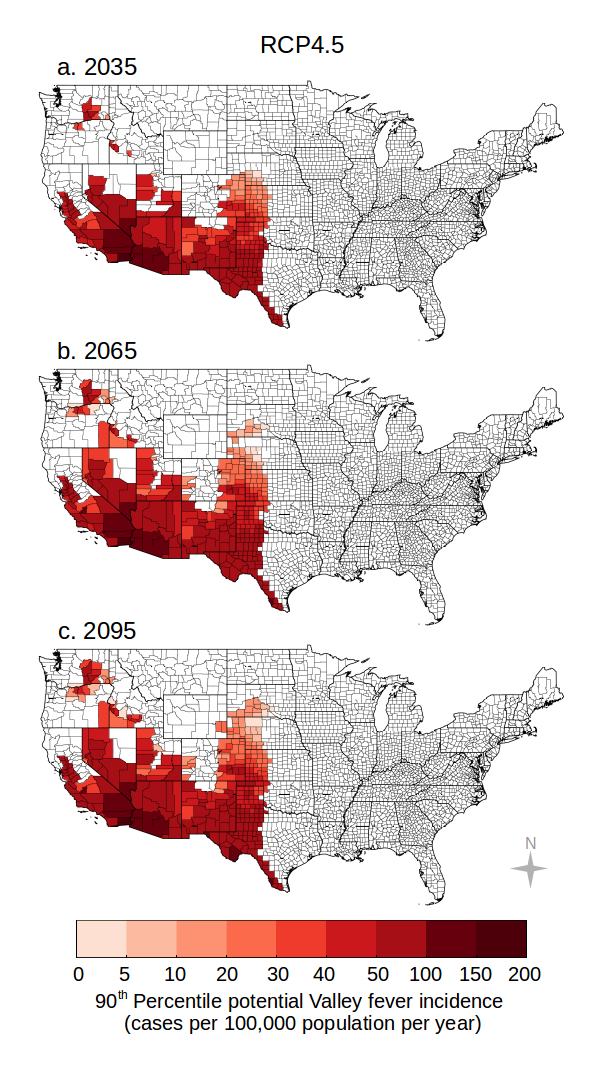
**

**Figure S5.** We estimated an upper bound of future Valley fever incidence using a 90th percentile regression model for (a) our 2007 baseline period, (b) 2035, (c) 2065, and (d) 2095 for RCP4.5. Over time, our model predicted Valley fever incidence will increase throughout the extreme southwestern US and the southern Great Plains. Incidence will also increase throughout the Central Valley of California and in the northwestern US.
